# Are prevention of mother-to-child HIV transmission service providers acquainted with national guideline recommendations? A cross-sectional study of primary health care centers in Lagos, Nigeria

**DOI:** 10.1186/s12913-022-08152-6

**Published:** 2022-06-11

**Authors:** B. Okusanya, C. Nweke, L. B. Gerald, S. Pettygrove, D. Taren, J. Ehiri

**Affiliations:** 1 Department of Health Promotion Sciences, Mel and Enid Zuckerman College of Public Health University of Arizona, Tucson, AZ USA; 2 Department of Nursing Science, Faculty of Clinical Sciences, College of Medicine, University of Lagos, Idi-Araba, Lagos, Nigeria; 3 Department of Epidemiology, Mel and Enid Zuckerman College of Public Health University of Arizona, Tucson, AZ USA; 4 Department of Pediatrics, School of Medicine, University of Colorado, Aurora, CO USA

**Keywords:** PMTCT, Guideline knowledge, eMTCT, Health workers knowledge

## Abstract

**Background:**

Implementation of interventions for the prevention of mother-to-child transmission (PMTCT) of HIV in low- and middle-income countries, faces several barriers including health systems challenges such as health providers’ knowledge and use of recommended guidelines**.** This study assessed PMTCT providers’ knowledge of national PMTCT guideline recommendations in Lagos, Nigeria.

**Methods:**

This was a cross-sectional survey of a purposive sample of twenty-three primary health care (PHC) centers in the five districts of Lagos, Nigeria. Participants completed a self-administered 16-item knowledge assessment tool created from the 2016 Nigeria PMTCT guidelines. Research Electronic Data Capture (REDCap) was used for data entry and R statistical software used for data analysis. The Chi square test with a threshold of *P* < 0.05 considered as significant was used to test the hypothesis that at least 20% of service providers will have good knowledge of the PMTCT guidelines.

**Results:**

One hundred and thirteen (113) respondents participated in the survey. Most respondents knew that HIV screening at the first prenatal clinic was an entry point to PMTCT services (97%) and that posttest counselling of HIV-negative women was necessary (82%). Similarly, most respondents (89%) knew that early infant diagnosis (EID) of HIV should occur at 6–8 weeks of life (89%). However, only four (3.5%) respondents knew the group counselling and opt-out screening recommendation of the guidelines; 63% did not know that haematocrit check should be at every antenatal clinic visit. Forty-eight (42.5%) service providers had good knowledge scores, making the hypothesis accepted. Knowledge score was not influenced by health worker cadre (*p* = 0.436), training(*P* = 0.537) and professional qualification of ≤5 years (*P* = 0.43).

**Conclusion:**

Service providers’ knowledge of the PMTCT guidelines recommendations varied. The knowledge of group counselling and opt-out screening recommendations was poor despite the good knowledge of infant nevirapine prophylaxis. The findings highlight the need for training of service providers.

**Supplementary Information:**

The online version contains supplementary material available at 10.1186/s12913-022-08152-6.

## Background

Globally, Nigeria has the second-largest burden of HIV infection [1]. Recent evidence shows that Nigeria has an adult HIV prevalence rate of 1.4% [2] and because of its large population of over 200 million [3], the country remains one of twenty-three priority countries identified by the World Health Organization (WHO) as accounting for 90% of pregnant women living with HIV globally [4]. Nigeria is responsible for 30% of the world’s gap [5] in achieving the global target of eradicating mother-to-child transmission (MTCT) of HIV. Although Nigeria has made notable progress in addressing the MTCT of HIV, significant challenges remain. For instance, in 2017: (i) only 35% of pregnant women who attended prenatal care were tested for HIV, (ii) only an estimated 30% of HIV-infected pregnant women received antiretroviral (ARV) therapy, and (iii) 36,000 children became HIV infected [4].

With an estimated 17.5 million people [3] and an HIV prevalence of 1.3%, [6] Lagos state contributes a large proportion of Nigeria’s new pediatric HIV infections. Given that Lagos is the state with the highest population in Nigeria, efforts to accelerate the use of PMTCT treatment guidelines in routine care are urgent. From the HIV prevalence of Lagos state in 2019, the state had an estimated 14,224 pregnant women living with HIV and 6400 preventable new pediatric HIV infections without PTMCT [2]. PMTCT reduces transmission of HIV from mother to child during pregnancy, labor, delivery, or breastfeeding from 45 to 2% [7]. Nigeria is not on target to eliminate new HIV infections among children and has the slowest decline in MTCT rate in sub-Saharan Africa [4]. In 2017, Nigeria had an MTCT rate of 26.9% despite the support of non-governmental organizations (NGOs) committed to fighting HIV/AIDS [4]. Nigeria is one of four countries with over 10,000 new pediatric HIV infections per annum [8]. MTCT may occur during pregnancy, labour, and after childbirth, with 10–15% of the highest risks during breastfeeding [9].

To address the high burden of HIV/AIDS in many low- and middle-income countries, the WHO established prevention and treatment guidelines to assure efficiency in resource use and better patient outcomes. PMTCT guidelines strategies starts with screening all pregnant women at their first prenatal clinic for HIV infection, and ends with providing an appropriate infant feeding option [9, 10]. It also highlights the need for repeat HIV screening of HIV-negative women in the third trimester of pregnancy or childbirth, depending on when their first prenatal clinic occurred [9, 10]. The current Nigerian PMTCT guidelines recommend the use of cART for life once diagnosed with HIV infection [9]. When compliance with PMTCT guidelines in routine care is optimal, the frequency rates of MTCT reduces to 2–5% from 25 to 40%, depending on the infant feeding option of the mother [9, 10].

To ensure proper implementation of PMTCT recommendations in routine care, the Nigerian government and non-governmental agencies have trained PMTCT providers on implementing the recommendations. However, their efforts have not been effective in reducing the frequency of MTCT [11]. Providers were trained that HIV-negative women and women with unknown HIV status should have counseling and testing for HIV, and ART coverage was significantly higher in “training-intervention” maternity units than control maternity units [12]. Yet, a systematic review of interventions to improve PMTCT service delivery and promote client retention reported the effect of health workers’ training as unclear [13]. While the treatment guidelines are based on scientific evidence and could help reduce pediatric HIV infections, research findings on health workers’ knowledge of the guidelines or implementation in routine practice are limited. For instance, providers participating in qualitative research in Tanzania reported partial and incomplete knowledge about PMTCT recommendations [14]. In an assessment of infant feeding practices in the context of HIV in four African countries, 70% of providers were unable to correctly estimate transmission risks of breastfeeding, irrespective of PMTCT training [15]. Also, providers in Malawi had limited knowledge of the timing of MTCT of HIV infection and the effectiveness of PMTCT interventions [16]. On the contrary, 83% of providers identified MTCT as the primary source of pediatric HIV infection, and 87% knew that HIV-positive women do not always transmit the disease to their children [17]. Good knowledge of PMTCT guidelines was influenced by the cadre of provider, application of knowledge during routine work, and length of service [18, 19].

The attainment of PMTCT treatment targets for Nigeria is crucial to that of the world. No studies have investigated providers’ knowledge of Nigeria’s PMTCT guidelines in routine practice. Providers’ knowledge of the PMTCT guidelines recommendations is key to implementing prescriptions in routine care and eventual reduction in MTCT rates in Nigeria. Therefore, providers of PMTCT services at the primary health care level should be well-acquainted with the most current PMTCT guideline prescriptions and prevailing evidence to care for pregnant women living with HIV. While knowledge alone does not translate into practice, it is a necessary first step in influencing outcomes. Therefore, the objective of this study was to assess whether health workers who provide PMTCT services in PHC centers in Lagos State, Nigeria, are familiar with the National program guideline recommendations.

## Methods

This cross-sectional self-administered survey assessed the knowledge of PMTCT guidelines recommendations by providers at the primary health care level in Lagos state. For this research, providers refer specifically to midwives, nurses, and doctors, the predominant cadres of PMTCT providers at PHC centers. Although medical records staff, laboratory technicians, and pharmacy technicians have specified tasks and roles in the PMTCT continuum of care, they were excluded from the research since they do not implement core PMTCT of HIV interventions. In addition, community health extension workers (CHEWs) were excluded because they do not work in the maternity units of PHC centers in Lagos state.

### Study approval and selection of participating health facilities

The first part of the survey tool was the electronic study information sheet. Participants read and had an opportunity to clarify concerns before providing written consent. Written informed consent was provided by all participants and captured electronically on RedCap before participants started the survey.

Participation in the study was voluntary, and no incentives were provided. Respondents had the right to decline participation or to withdraw from the study at any stage if they wished. Ethical approvals for the study were obtained from the Human Health Research Ethics Committee of the Lagos University Teaching Hospital (ADM/DCST/HREC/ APP/4031) and the Institutional Review Board (IRB) of the University of Arizona, USA (Protocol Number: 2101422001). All methods were carried out in accordance with relevant guidelines and regulations.

**Fig. 1:**
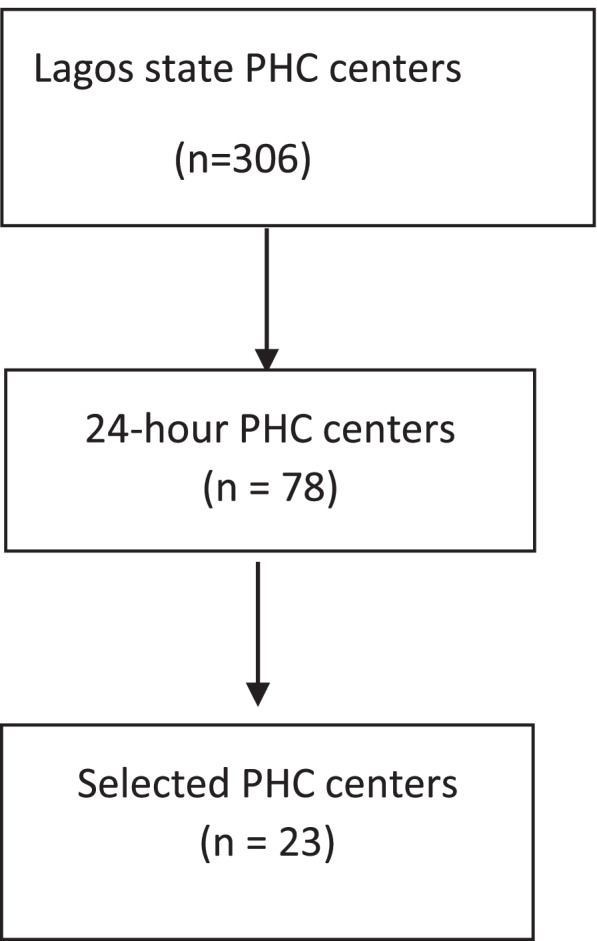
Health facility selection process

The Lagos State Primary Health Care Board (LSPHCB) approved the research and provided a comprehensive list of PHC centers that offer 24-hour services in the state. There were 78 PHC centers offering 24-hour services of the 306 PHC centers in Lagos State. Figure 1 shows the selection of 23 participating PHCs. LSPHB also provided the research team with a count of pregnant women living with HIV infection who had PMTCT interventions and the number of people (including males) living with HIV infection who received care at its facilities in 2020. Purposive selection of high HIV prevalent local government areas (LGAs) to participate in the study was done using the lists. Selected LGAs reported 83–1281 new HIV infections in 2020 and cared for 9–121 pregnant HIV-infected women in the same year. Also, the selected PHC centers are spread across the five administrative districts of Lagos State. The facility selection was based on the assumption that care provided to people living with HIV will enhance the knowledge of treatment guidelines as reported in a publication [19].

**Fig. 2:**
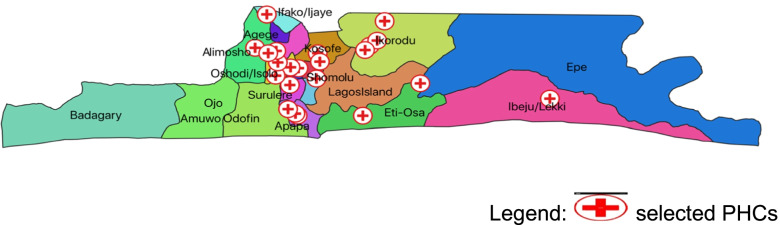
Location map of included primary health centers

Twenty-three (23) PHC centers were selected to participate. Figure 2 shows the location and spread of the PHCs across the state. Within each PHC, consecutive enrollment of service providers was used to achieve the minimum desired sample size of 109 as calculated for the project. The sample size of 109 respondents was calculated using STATA software and a one-sided, one proportion test, assuming that a minimum of 20% of providers will have good PMTCT knowledge to test a difference in knowledge of 10% at a power of 0.8.

### Respondents

Respondents were nurses, midwives, or doctors who provided PMTCT services at participating PHC centers in Lagos state. These health workers offered prenatal care, screened pregnant women for HIV infection, and supervised childbirth, including newborn care. At each PHC centers, the researchers approached PMTCT providers after the facility’s officer-in-charge (OIC) permitted entry into the facility. To facilitate facility entry, the approval from the Lagos State Primary Health Board was communicated to the Medical Officer of Health (MoH) of the respective local government area (LGAs). The MoH, in turn, informed the OICs of the facilities. To ensure fieldwork occurred on a mutually convenient day, telephone calls were made to each OIC ahead of the visit and to build rapport with them and to know the most convenient day of the week and time for their research participation. All respondents participated in the research during working days except one conducted on a Saturday at the convenience of the service provider. For this research, participants were recruited one after the other based on availability or convenience to participate in the research at the maternity units of participating health facilities until the sample size was achieved. Although few providers have been reported to work at PHC centers in Lagos state [20], this approach was pragmatic. Logistical challenges were reduced by limiting the number of health facilities to 23 to attain the targeted sample size.

Knowledge assessment: This was the primary outcome of the research. Although many previous studies on PMTCT knowledge assessed one or other PMTCT interventions, this study evaluated knowledge of each PMTCT strategy. Also, early infant diagnosis of HIV infection, childhood immunization, and opportunistic infection prophylaxis for the HIV-exposed infant knowledge assessment were included. A 16-item knowledge-assessment tool (Appendix 1), created from the Nigerian 2016 National Consolidated HIV Treatment Guidelines for PMTCT [9] was used. The National PMTCT Task Team of the Federal Ministry of Health of Nigeria developed the guidelines as an adaptation of the 2016 WHO PMTCT treatment guidelines [10]. The research hypothesis was that at least 20% of providers in Lagos State, Nigeria, would have good knowledge of PMTCT guidelines by achieving more than 50% on the PMTCT guideline knowledge scale. The knowledge score scale was categorized as 1). Good (9–16 correct answers), 2). Poor (≤8 correct answers). Experts reviewed all questions in the research tool for face and content validity before field-testing was conducted in two PHC centers by ten service providers not included in the main study. Results of the pilot test were used to revise the content and wording of the instrument. Study covariates include participant’s age, health worker’s cadre, number of PMTCT clients attended per month, number of years of professional qualification, previous PMTCT training and interval from the last PMTCT training to the study. Data were collected from July 28 to September 10 2021 and managed using REDCap [21, 22].

### Statistical analysis

Data analyses were performed using R statistical software. Normality tests for continuous variables were performed with the Shapiro-Wilk’s test, and skewness was assessed with Kurtosis. The continuous variables were not normally distributed. The two missing values for the number of professional years of qualification was replaced with the median value. Tests of statistical association of categorical variables were conducted, using Chi-square test with a level of significance of < 0.05. All nurses and midwives were categorized as a single group and compared with doctors. The years of professional qualification was categorized as ≤5 years (recent) and ≥ 6 years (not recent) for tests of association. Also, the interval from last PMTCT training to knowledge assessment was categorized as ≤5 years (recent) and ≥ 6 years, and then as ≤2 years (recent) and ≥ 3 years (not recent) for tests of association. The ggplot function of R was used to present boxplots. The Global Positioning System (GPS) coordinates of participating PHC centers were used to show the spatial location of participating PHCs centers and the knowledge score of its health workers.

## Results

### Characteristics of respondents

Characteristics of respondents are shown in Table 1. One hundred and thirteen health workers in 23 PHC centers participated in the study. Respondents were Nurses/Midwives (53.0%; *n* = 60), Midwives (20.4%; *n* = 23), Nurses (7.1%; *n* = 8) and doctors (19.5%; *n* = 22). Their ages ranged from 22 to 59 years, with median and inter-quartile range of 41 (18) years. Doctors who participated in the study were younger (36.3 ± 4.1 yrs.) than the nurses (43.1 ± 10.9 yrs.), midwives (35 ± 10.6 yrs.) and Nurses & Midwives (47.4 ± 9 yrs.).

**Table 1:** Respondents’ characteristics

Respondents’ characteristics	Number	Percentage
Age (years)		
20–30	14	12.4
31–40	42	37.2
41–50	23	20.4
51–60	34	30.0
Ethnicity		
Yoruba	91	80.5
Igbo	4	3.5
EDO	3	2.7
Ibibio	2	1.8
Others	4	3.5
Unspecified	9	8.0
Religion		
Christianity	90	79.6
Islam	23	20.4
Marital status		
Married	89	78.8
Single	17	15.0
Widow	7	6.2
Gender		
Females	98	86.7
Males	14	12.4
Unspecified	1	0.9
Previous PMTCT training		
Yes	69	61.1
No	40	35.4

The majority of respondents were females (86.7%; *n* = 98), with males accounting for 12.4% (*n* = 14) and one respondent not identifying with either gender. Most respondents had Yoruba ethnicity (80.5%; *n* = 91). The ethnicity of other respondents were Igbo (3.5%; *n* = 4), Edo (2.7%; *n* = 3), Ibibio (1.8%; *n* = 2), others (3.5%; *n* = 4) and 7.9% (*n* = 9) did not provide their ethnicity.

The number of years since the professional qualification of respondents ranged from 1 to 52 years, with a median of 12 (IQR = 20) years. Respondents attended to a median of 2 (IQR = 2) pregnant women living with HIV infection per month. Sixty-nine (*n* = 69) respondents had received training on PMTCT interventions, and 38.9% (*n* = 44) had not. While the median age of respondents who had PMTCT training was 47 (IQR = 18) years, that of respondents who had no training was 35.5 (IQR = 12.5) years. The median interval between their last training and knowledge assessment was 39.3 (IQR = 60) months for those trained. While a government agency trained 42% (*n* = 29/69), 58% (*n* = 40) were trained by non-governmental organizations implementing PMTCT interventions in the state, with most of the training (56/69; 81.1%) held outside of the health facilities. More nurses/midwives (65%; 59/91) had PMTCT training than doctors (45.5%; 10/22). There was no statistically significant association between the cadre of health workers and PMTCT training (X^2^ = 2.04; df = 1; *P* = 0.153).

### Knowledge of guidelines recommendations

Respondents’ knowledge of PMTCT guideline recommendations is presented in Table 2. Most respondents (97%) knew that HIV screening at the first prenatal clinic is an entry point to PMTCT services and that posttest counselling of HIV-negative women is necessary (82%). Also, most respondents (97%) knew that nevirapine prophylaxis should start within 72 hours of birth, and early infant diagnosis of HIV infection by polymerase chain reaction should be at 6–8 weeks of life (89%). On the contrary, only 3.5% (*n* = 4) of respondents knew that the PMTCT guidelines recommend group counselling and opt-out screening of HIV in pregnancy. Most respondents (62.8%; *n* = 71) did not know that the packed cell volume (PCV) of pregnant women living with HIV should be checked at the first and at each subsequent antenatal clinic visit. Also, only 38% (*n* = 43) knew that discussion of postpartum family planning with pregnant women living with HIV should not start at the postnatal clinic after birth, but during the prenatal period.

**Table 2:** Respondents’ knowledge of PMTCT EID guideline recommendations

PMTCT Intervention Domain	Items on knowledge assessment tool	Respondents’ response	
		Correct (%)	Incorrect (%)
Provision of integrated PMTCT services within routine prenatal care	The PMTCT guidelines recommend: a). Group counselling and Opt-in HIV testing b). Individual counselling and opt-out testing c). Group counselling and opt-out testing	4 (3.5)	109 (96.5)
	Screening of HIV at the “Booking clinic’ is the entry into PMTCT services	109 (96.5)	4 (3.5)
Screening of all pregnant women at their first prenatal clinic for HIV infection	Posttest counselling of HIV-negative women is not required	193 (82.3)	20 (17.7)
Screening of all HIV positive pregnant women for co-morbid opportunistic infections like Tuberculosis	You should check the blood levels (PCV) of a pregnant HIV positive woman: a). at the booking clinic and every other clinic b). at the booking clinic and 3 other clinics c). at the booking clinic and whenever it is necessary	42 (37.8)	71 (62.8)
	HIV viral load in pregnancy should be checked: a). At the booking clinic only b). At the booking clinic and 34–36 weeks’ gestation c). At the booking visit, 34–36 weeks’ gestation and in labour	60 (53.1)	53 (46.9)
Provision of posttest counselling to all women and link all HIV-positive women to ART initiation	Select the wrong sentence from below: a). HIV medications once started in pregnancy should be used for life. b). The preferred medication is (Tenofovir/Lamivudine/Efavirenz) known as Telura® c). Not all HIV positive pregnant women need to take HIV medications	78 (69)	33 (29.2)
Performance of repeat HIV screening for HIV-negative women late in pregnancy and intrapartum.	A woman who had an HIV-negative test result at 24 weeks should have a repeat HIV test at 34–38 weeks’ gestation	91 (80.5)	22 (19.5)
	A woman who had an HIV-negative test result at 18 weeks should have a repeat HIV test at 34–38 weeks’ gestation	90 (79.6)	23 (20.4)
Intrapartum interventions	Artificial rupture of fetal membranes (ARM) should be performed in labour when the cervix is ≥7 cm dilated	43 (38.0)	70 (62.0)
	Episiotomy (surgical perineal cut) should be given to make HIV positive women deliver quicker.	96 (85.0)	17 (15.0)
Commencement of ART infant prophylaxis to all HIV-exposed infants within 72 hours of birth	All HIV-exposed infants should have Nevirapine syrup daily soon after birth, within 72 hours.	109 (96.5)	2 (1.8)
	A high-risk HIV-exposed infant has a mother: a. Who used ART for more than 4 weeks at the time of birth. b. Who has a viral load > 1000 copies/ml 4 weeks before birth. c. All of the above	60 (53.1)	52 (46.0)
Maternal postpartum care	Discussion of postpartum family planning should start at the postnatal clinic.	43 (38.0)	70 (62.0)
Childhood immunization for the HIV-exposed infant	HIV-exposed infants should not be given the second dose of the Oral Polio vaccine at 6 weeks old.	104 (92.0)	9 (8)
Early infant HIV diagnosis (EID)	Dry blood sample (DBS) test (DNA PCR) is done at 6–8 weeks of life for HIV-exposed infant	100 (88.5)	12 (10.6)
Opportunistic infection prophylaxis	Cotrimoxazole (Septrin®) prophylaxis for all HIV-exposed infants at 6 weeks is not necessary.	82 (72.6)	31 (27.4)

Items without options to choose from were answered True or False

 Fifty (44.2%), and 63 (55.8%) respondents, respectively, had good and fair knowledge scores of the recommendations of the PMTCT guidelines. The distribution of the score categories across LGAs is as presented in Table 4. More participants had good knowledge score in only one LGA, while in 3 other LGAs 50% of participants had either good or poor scores. Participants with poor scores were more in the remaining four LGAs. There was no statistically significant association between the performance on the knowledge assessment tool and cadre of health workers (X^2^ = 0.61, *P*-value = 0.436), previous PMTCT training (X^2^ = 0.38; *P* = 0.537), and professional qualification of 5 years or less (X^2^ = 0.622; *P* = 0.43). When the interval of PMTCT training to knowledge assessment was categorized as recent ≤5 years, and then as ≤2 years there was no significant association between the performance on the knowledge assessment and training within 5 years (X^2^ = 0.86; *P* = 0.651), or within 2 years (X^2^ = 0.83; *P* = 0.66) of the study; see Table 3 for the knowledge scores covariates. Table 4 shows the distribution of the knowledge score across LGAs.

**Table 3:** Knowledge scores covariates

Co-variates	Knowledge assessment performance		*P*-value
	Good	Poor	
Service providers cadre			
Nurses	70	21	0.436
Physicians	14	8	
Previous PMTCT training			
Trained	51	18	0.537
Untrained	33	11	
Professional qualification < 5 years			
≤5 years	19	2	0.430
> 5 years	63	27	
PMTCT training interval to survey < 5 years			
≤5 years	31	11	0.651
> 5 years	20	7	
PMTCT training interval to survey < 2 years			
≤ 2 years	21	5	0.662
> 2 years	30	14

**Table 4:** Distribution of knowledge score categories across local government areas (LGAs)

LGAs	Knowledge score categories		
	Good N (%)	Poor N (%)	Number of LGA Participants
Ajeromi	7 (50)	7 (50)	14
Alimoso	10 (53)	9 (47)	19
Ifako-Ijaiye	4 (33)	8 (67)	12
Eti-Osa LGA	6 (50)	6 (50)	12
Ikorodu LGA	10 (50)	10 (50)	20
Oshodi-Isolo	8 (40)	12 (60)	20
Kosofe	4 (33)	8 (67)	12
Shomolu	1 (25)	3 (75)	4
Total knowledge for score category	50 (44.2)	63 (55.8)	113

## Discussion

The implementation of global and national PMTCT guidelines and recommendations is met with numerous challenges. Although resource limitation has been cited as a significant barrier against effective program implementation in low- and middle-income countries, health providers’ knowledge of practice guidance, which is the first step in program implementation, is an often-neglected health system barrier.

In this study, 44% of respondents had good knowledge of the recommendations of the PMTCT guidelines. This confirms the research hypothesis that at least 20% of providers working at participating PHCs in Lagos have good knowledge scores. Sixty per cent of respondents have had PMTCT training within 3 years prior to the survey. The research findings have tremendous implications for the practice of PMTCT services at the PHC level of Lagos state. For instance, only four (3.5%) respondents knew the group counselling guidelines and opt-out screening recommendation. In settings where group counselling and testing for HIV infection in pregnancy was introduced after opt-in testing had been used, there was a significant increase in the uptake of testing with group testing and opt-out screening [23, 24]. There is a possibility that the respondents’ response was influenced by their practice rather than by the guidelines. Also, compared with their HIV-uninfected counterparts, pregnant women living with HIV infection are at an increased risk of anemia in pregnancy [25, 26]. The knowledge assessment indicated that more than half of respondents did not know that packed cell volume/ haematocrit check should be at every antenatal clinic visit for pregnant women living with HIV infection. This portends missed opportunities for early detection of anemia in pregnancy, leading to other pregnancy complications like intrauterine growth restriction, especially in women living with HIV infection.

One of the critical elements of PMTCT interventions is the use of effective contraceptives by women living with HIV infection to prevent unintended pregnancy or for child spacing. For this to be effective, women living with HIV in pregnancy should have prenatal discussions on family planning. Most respondents indicated that postpartum family planning discussions should start at the postnatal clinic 6 weeks after birth. This inaccurate knowledge might explain the low postpartum contraceptive usage amongst women with HIV infection and affirms earlier reports of missed opportunities for contraceptive counselling for women with HIV [27]. For instance, 37.9% of women living with HIV in pregnancy missed family planning counselling during prenatal care, and 24% of the women did not have any family planning counselling during the provision of PMTCT services [27]. When provided, 34% of women living with HIV infection had prenatal family planning counselling [28]. The Nigerian demographic and health survey 2018 reported a low contraceptive prevalence rate of 17%, with the need to improve the use of contraceptives, including by women living with HIV infection [29].

The recommendation to repeat antenatal HIV screening for pregnant women whose first screening test was negative was known by most respondents. The treatment guideline recommends that women whose first HIV screening test was earlier than 28 weeks’ gestation have a repeat antenatal screening test [9, 10]. The knowledge of this recommendation is likely to reduce needless repeat HIV screening in pregnancy and perhaps a waste of screening kits and consumables within the facilities. This has implications on the availability of HIV screening items at the primary level of care in the state. In settings where test and retest of HIV in pregnancy had been assessed, only 11% of eligible women had two prenatal HIV screening [30]. Forty-three (43%) per cent of respondents had good knowledge of the recommendations of the PMTCT guidelines, and each participant attended to two pregnant women living with HIV infection every month. Aside from achieving the research hypothesis, these findings also imply that there might not be enough clients to stimulate guidelines knowledge retention as attending to HIV-infected women regularly has been reported to improve guideline knowledge retention [19]. The fact that 60% of respondents had training, with a training interval of about three years, might also explain the knowledge score across the study because knowledge reduces over time [19], and the score categories include scores of service providers with no training.

More often, knowledge of PMTCT guidelines alone does not translate into practice, and the quality of PMTCT services may be influenced by the presence of an adequate number of trained providers [31]. Other health system challenges identified in PMTCT service provision in Nigeria include women’s suboptimal uptake of antenatal services of 67%, and the lack of national PMTCT unique identification systems to enhance quality care [5, 29]. In addition, continuity of care is an issue because when people change residence, their medical records are not transferred to the new cART treatment facility. Hence, their health records are inaccessible to their new provider because they are enrolled all over in the PMTCT program and may be counted twice at the national programmatic level. Other identified barriers include resource constraints [32] lack of national guidelines documents, weak planning for guidelines dissemination, and lengthy guidelines. The challenges of the rigour of performing some detailed guidelines activities (like the WHO clinical staging of HIV disease), staff availability, and lack of supplies also exist [33]. Moreover, as new evidence became available, the frequent changes in PMTCT guideline recommendations in the last decade might hinder PMTCT guideline use in routine care. Specifically, PMTCT guidelines were regularly updated every 3 years as new evidence became available [10, 34, 35]. Some of these changes include the use of combined antiretroviral therapy (cART), formerly called highly active retroviral therapy (HAART) introduced in 2009, and the addition of efavirenz as a first-line medication in 2013 when safety in pregnancy was confirmed [34, 35]. In 2016, the “test and treat” strategy and use of cART for life were recommended [10]. The 2018 updated recommendations on first-line and second-line antiretroviral regimens and post-exposure prophylaxis and recommendations on early infant diagnosis of HIV have been adopted in Nigeria, with Dolutegravir (DTG) and Raltegravir (RAL) now prescribed in pregnancy [36]. The frequent treatment guidelines update may lead to insufficient knowledge of health workers [32] and be a challenge to translating PMTCT guidelines into routine care [37].

PMTCT interventions are a spectrum of care during pregnancy, childbirth and postpartum [9, 10]. Previous studies that assessed health workers’ knowledge of PMTCT services had focused on select aspects of the spectrum of care. The strength of this study includes the comprehensive assessment of providers’ knowledge on prenatal, intrapartum, and postpartum recommendations of the National PMTCT guidelines for Nigeria. Assessing providers’ knowledge at the primary health care level, the most proximal level of care to the people is another strength. The selection of participating health facilities purposively based on LGAs with a high prevalence of people living with HIV, including pregnant women in the preceding year to the study, ensured the selection of facilities with good patronage for PMTCT services at the primary health care system in the state. This approach increased the likelihood of involving health workers with good knowledge of PMTCT guideline recommendations. However, this might have biased the sample toward having a greater per cent of service providers who know the recommendations, thereby overestimating the reported knowledge scores. Importantly, selected sites were across the five administrative districts of Lagos State, making the findings more representative of knowledge of PMTCT service providers at the state’s primary health care level. That said, the study is limited by involving health workers at only one level of care, making its findings not generalizable to the state’s health system, or indeed, that of other states. Also, other factors required for proper PMTCT guidelines implementation, such as availability of resources and conducive work environment, were not assessed in this study. Despite these limitations, the results represent the situation at participating health facilities.

With dwindling funding for HIV infection prevention, offsite PMTCT training held for many years with the PEPFAR (President’s Emergency Plan for AIDS Relief) interventions is becoming less often. The need for PMTCT educational resources delivered electronically over the web or with the use of pre-loaded software that health workers can use to learn about PMTCT should be considered. Self-paced training in modules and knowledge assessment on interactive electronic platforms may be used. This will provide health workers with a chance to review their answers to questions during the training. When maternal, newborn and child health (MCH) educational contents were delivered electronically using preloaded videos on tablets to frontline health workers in rural Nigeria, health workers knowledge improved, and they became motivated and confident to render health care services to their clients [38]. Hence, pre-loaded PMTCT-training contents on tablets as a learning model for ongoing PMTCT training of health workers should be considered. This is because many health workers are familiar with smartphones and would find the training useful and acceptable [38].

## Conclusion

Assessing the health systems’ challenges, including providers’ knowledge of the recommendations of PMTCT guidelines, is essential to the eventual attainment of zero perinatal transmission of HIV infection. Findings that only 4 out of every ten participating health workers had good knowledge of the guideline’s recommendations imply training needs. Regular training either in person or remotely with preloaded educational resources on PMTCT services is needed for proper and sustained program implementation to attain zero perinatal HIV transmission.

## Supplementary Information

**Additional file 1: Appendix.** Questions for knowledge assessment of PMTCT providers.

## Data Availability

The datasets generated and analyzed during the current study are not publicly available because they will be used for secondary analysis and more publications in the near future but are available from the corresponding author on reasonable request.

## Abbreviations

ART: Antiretroviral therapy
cART: Combined antiretroviral therapy
CHEWs: Community health extension workers
DTG: Dolutegravir
EID: Early infant diagnosis
GPS: Global positioning systems
HAART: Highly active antiretroviral therapy
HEIs”: HIV-exposed infants
HIV/AIDS: Human immune deficiency virus/ acquired immune deficiency syndrome
IRB: Institutional review board
LSPHB: Lagos state primary health board
LGAs: Local government areas
MoH: Medical officer of health
MTCT: Mother-to-child-transmission
OIC: Officer-in-charge of facility
PEPFAR: President’s Emergency Plan For AIDS Relief
PHC: Primary health care
PMTCT: Prevention of mother-to-child-transmission
RAL: Raltegravir
REDCap: Research electronic data capture
WHO: World Health Organization

## References

[1] Avert (2019). HIV and AIDS in Nigeria.

[2] Federal Ministry of Health Abuja N (2019). Nigeria National HIV/AIDS indicator and impact survey (NAIIS).

[3] Review WP (2019). Nigeria population 2019.

[4] Avert (2019). Prevention of mother-to-child transmission (Pmtct) of HIV.

[5] Oladele EA, Khamofu H, Asala S, Saleh M, Ralph-Opara U, Nwosisi C, Anyaike C, Gana C, Adedokun O, Dirks R (2017). Playing the catch-up game: accelerating the scale-up of prevention of mother-to-child transmission of HIV (PMTCT) services to eliminate new pediatric HIV infection in Nigeria. PLoS One.

[6] National Agency for the control of AIDS (NACA) Abuja N (2019). Nigeria prevalence rate.

[7] Luzuriaga K, Mofenson LM (2016). Challenges in the elimination of pediatric HIV-1 infection. N Engl J Med.

[8] UNAIDS (2018). Miles to go closing gaps breaking barriers righting injustice.

[9] National AIDS and STI’s Control Programme FMoHAN (2016). National guidelines for HIV prevention treatment and care.

[10] Organization WH (2016). Consolidated guidelines on the use of antiretroviral drugs for treating and preventing HIV infection. Recommendations for a public health approach.

[11] Burlew R, Puckett A, Bailey R, Caffrey M, Brantley S (2014). Assessing the relevance, efficiency, and sustainability of HIV/AIDS in-service training in Nigeria. Hum Resour Health.

[12] Kieffer MP, Nhlabatsi B, Mahdi M, Hoffman HJ, Kudiabor K, Wilfert CM (2011). Improved detection of incident HIV infection and uptake of PMTCT services in labor and delivery in a high HIV prevalence setting. J Acquir Immune Defic Syndr.

[13] Ambia J, Mandala J (2016). A systematic review of interventions to improve prevention of mother-to-child HIV transmission service delivery and promote retention. J Int AIDS Soc.

[14] Shayo EH, Vaga BB, Moland KM, Kamuzora P, Blystad A (2014). Challenges of disseminating clinical practice guidelines in a weak health system: the case of HIV and infant feeding recommendations in Tanzania. Int Breastfeed J.

[15] Chopra M, Rollins N (2008). Infant feeding in the time of HIV: rapid assessment of infant feeding policy and programmes in four African countries scaling up prevention of mother to child transmission programmes. Arch Dis Child.

[16] Kafulafula UK, Hutchinson MK, Gennaro S, Guttmacher S (2014). Maternal and health care workers' perceptions of the effects of exclusive breastfeeding by HIV positive mothers on maternal and infant health in Blantyre, Malawi. BMC Pregnancy Childbirth.

[17] Djadou KE, Koffi KS, Saka B, Tepe EM, Vinyo DK, Tatagan-Agbi K (2011). Knowledge, attitudes and practices of healthcare providers in Togo regarding prevention of mother-to-child transmission of HIV in 2010. Med Trop.

[18] Paul T, Marie TP, Bechem E (2017). Knowledge, attitude and practice of staff of 4 hospitals in Yaounde on the prevention of vertical transmission of hepatitis B. Pan Afr Med J.

[19] Kufe NC, Metekoua C, Nelly M, Tumasang F, Mbu ER (2019). Retention of health care workers at health facility, trends in the retention of knowledge and correlates at 3rd year following training of health care workers on the prevention of mother-to-child transmission (PMTCT) of HIV-National Assessment. BMC Health Serv Res.

[20] Monica Das Gupta MD GV, Khemani S (Development Research Group). The World Bank: Decentralized Delivery of Primary Health Services in Nigeria. Survey Evidence from the States of Lagos and Kogi. 2003.

[21] Harris PA, Taylor R, Minor BL, Elliott V, Fernandez M, O'Neal L, McLeod L, Delacqua G, Delacqua F, Kirby J (2019). The REDCap consortium: building an international community of software platform partners. J Biomed Inform.

[22] Harris PA, Taylor R, Thielke R, Payne J, Gonzalez N, Conde JG (2009). Research electronic data capture (REDCap)--a metadata-driven methodology and workflow process for providing translational research informatics support. J Biomed Inform.

[23] Baisley K, Doyle AM, Changalucha J, Maganja K, Watson-Jones D, Hayes R, Ross D (2012). Uptake of voluntary counselling and testing among young people participating in an HIV prevention trial: comparison of opt-out and opt-in strategies. PLoS One.

[24] Chandisarewa W, Stranix-Chibanda L, Chirapa E, Miller A, Simoyi M, Mahomva A, Maldonado Y, Shetty AK (2007). Routine offer of antenatal HIV testing ("opt-out" approach) to prevent mother-to-child transmission of HIV in urban Zimbabwe. Bull World Health Organ.

[25] Dairo MD, Lawoyin TO, Onadeko MO, Asekun-Olarinmoye EO, Adeniji AO (2005). HIV as an additional risk factors for anaemia in pregnancy: evidence from primary care level in Ibadan, southwestern Nigeria. Afr J Med Med Sci.

[26] Muhangi L, Woodburn P, Omara M, Omoding N, Kizito D, Mpairwe H, Nabulime J, Ameke C, Morison LA, Elliott AM (2007). Associations between mild-to-moderate anaemia in pregnancy and helminth, malaria and HIV infection in Entebbe, Uganda. Trans R Soc Trop Med Hyg.

[27] Nabirye J, Matovu JKB, Bwanika JB, Makumbi F, Wanyenze RK (2020). Missed opportunities for family planning counselling among HIV-positive women receiving HIV Care in Uganda. BMC Womens Health.

[28] du Plessis E, Shaw SY, Gichuhi M, Gelmon L, Estambale BB, Lester R, Kimani J, Avery LS (2014). Prevention of mother-to-child transmission of HIV in Kenya: challenges to implementation. BMC Health Serv Res.

[29] ICF NPCNNa (2019). Nigeria demographic and health survey 2018.

[30] de Beer S, Kalk E, Kroon M, Boulle A, Osler M, Euvrard J, Timmerman V, Davies MA (2020). A longitudinal analysis of the completeness of maternal HIV testing, including repeat testing in Cape Town, South Africa. J Int AIDS Soc.

[31] Rowan BH, Robinson J, Granato A, Bla CK, Kouyate S, Djety GV, Abo K, Kone A, Gloyd S (2018). Workforce patterns in the prevention of mother to child transmission of HIV in cote d’Ivoire: a qualitative model. Hum Resour Health.

[32] Mulenga C, Naidoo JR (2017). Nurses’ knowledge, attitudes and practices regarding evidence-based practice in the prevention of mother-to-child transmission of HIV programme in Malawi. Curationis.

[33] Mwangome MN, Geubbels E, Wringe A, Todd J, Klatser P, Dieleman M (2017). A qualitative study of the determinants of HIV guidelines implementation in two south-eastern districts of Tanzania. Health Policy Plan.

[34] Organization WH (2009). Rapid advice antiretroviral therapy for HIV infection in adults and adolescents.

[35] Organization WH (2013). Consolidated guidelines on the use of antiretroviral drugs for treating and preventing HIV infection.

[36] Organization WH (2018). Updated recommendations on first-line and second-line antiretroviral regimens and post-exposure prophylaxis and recommendations on early infant diagnosis of HIV: Interim guidance.

[37] Laar AK, Amankwa B, Asiedu C (2014). Prevention-of-mother-to-child-transmission of HIV Services in sub-Saharan Africa: a qualitative analysis of healthcare providers and clients challenges in Ghana. Int J MCH AIDS.

[38] Ebenso B, Okusanya B, Okunade K, Akeju D, Ajepe A, Akaba GO, Yalma RM, Dirisu O, Tukur J, Abdullahi MK (2021). What are the contextual enablers and impacts of using digital technology to extend maternal and child health services to rural areas? Findings of a qualitative study from Nigeria. Front Glob Womens Health.

