# Supplementary material for: Are prevention of mother-to-child HIV transmission service providers acquainted with national guideline recommendations? A cross-sectional study of primary health care centers in Lagos, Nigeria
Source: BMC Health Serv Res. 2022 Jun 11;22:769. doi: 10.1186/s12913-022-08152-6 (PMC9188152; doi:10.1186/s12913-022-08152-6)
Supplement: Supplementary file 1 — Additional file 1: Appendix. Questions for knowledge assessment of PMTCT providers. [file 12913_2022_8152_MOESM1_ESM.docx]

Appendix: Questions for knowledge assessment of PMTCT providers

| Serial No: | PMTCT domain | Proposed Questions |
| --- | --- | --- |
|  | Provision of integrated PMTCT services within routine prenatal care | 1. The PMTCT guidelines recommend:   a). Group counselling and Opt-in HIV testing  b). Individual counselling and opt-out testing  c). Group counselling and opt-out testing |
|  |  | 1. Screening of HIV at the “Booking clinic’ is the entry into PMTCT services   a). True  b). False |
| 2. | Screening of all pregnant women at their first prenatal clinic for HIV infection | 1. Post-test counselling of HIV-negative women is not required   a). True  b). False |
| 3. | Screening of all HIV positive pregnant women for co-morbid opportunistic infections like Tuberculosis | 1. You should check the blood levels (PCV) of a pregnant HIV positive woman:   a). at the booking clinic and every other clinic  b). at the booking clinic and 3 other clinics  c). at the booking clinic and whenever it is necessary |
|  |  | 1. HIV viral load in pregnancy should be checked:   a). At the booking clinic only  b). At the booking clinic and 34-36 weeks gestation  c). At the booking visit, 34-36 weeks gestation and in labour |
| 4. | Provision of post-test counselling to all women and link all HIV-positive women to ART initiation | 1. Select the wrong sentence from below:   a). HIV medications once started in pregnancy should be used for life.  b). The preferred medication is (Tenofovir/Lamivudine/Efavirenz) known as Telura ®  c). Not all HIV positive pregnant women need to take HIV medications |
| 5. | - Performance of repeat HIV screening for HIV-negative women whose first prenatal clinic was < 28 weeks in the 3^rd^ trimester of pregnancy.  - Performance of repeat HIV screening for HIV-negative women whose first prenatal clinic was >28 weeks during childbirth. | 1. A woman who had an HIV-negative test result at 24 weeks should have a repeat HIV test at 34-38 weeks gestation   a). True  b). False |
|  |  | 1. A woman who had an HIV-negative test result at 18 weeks should have a repeat HIV test at 34-38 weeks gestation   a). True  b). False |
| 6. | Intrapartum interventions | 1. Artificial rupture of fetal membranes (ARM) should be performed in labour when the cervix is ≥ 7cm dilated.   a). True  b). False |
|  |  | 1. Episiotomy (surgical perineal cut) should be given to make HIV positive women deliver quicker.   a). True  b). False |
| 7. | Commencement of ART infant prophylaxis to all HIV-exposed infants within 72 hours of birth | 1. A high-risk HIV-exposed infant has a mother: 2. Who used ART for more than 4 weeks at the time of birth. 3. Who has a viral load >1000 copies/ml 4 weeks before birth. 4. All of the above |
|  |  | 1. All HIV-exposed infants should have Nevirapine syrup daily soon after birth, within 72 hours.   a). True  b). False |
| 8. | Maternal postpartum care | 1. Discussion of post-partum family planning should start at the postnatal clinic.   a). True  b). False |
| 9. | Childhood immunization for the HIV-exposed infant | 1. HIV-exposed infants should not be given the second dose of the Oral Polio vaccine at 6 weeks old.   a). True  b). False |
| 10. | Early infant HIV diagnosis (EID) | 1. Dry blood sample (DBS) test (DNA PCR) is done at 6-8 weeks of life for HIV-exposed infant   a). True  b). False |
| 11. | Opportunistic infection prophylaxis | 1. Cotrimoxazole (Septrin ®) prophylaxis for all HIV-exposed infants at 6 weeks is not necessary.   a). True  b). False |
